# Haulage Cycles Identification for Wheeled Transport in Underground Mine Using Neural Networks

**DOI:** 10.3390/s23031331

**Published:** 2023-01-25

**Authors:** Artur Skoczylas, Artur Rot, Paweł Stefaniak, Paweł Śliwiński

**Affiliations:** 1 KGHM Cuprum Research and Development Centre Ltd., Gen W. Sikorskiego 2-8, 53-659 Wroclaw, Poland; 2 Faculty of Management, Wroclaw University of Economics and Business, Komandorska 118/120, 53-345 Wroclaw, Poland; 3 KGHM Polska-Miedź S.A., M. Skłodowskiej-Curie 48, 59-301 Lubin, Poland

**Keywords:** ore transportation, artificial intelligence, underground mine, wheeled transport, operational regimes, neural networks, hyperparameters optimization

## Abstract

The task of ore transportation is performed in all mines, regardless of their type (open pit/underground) or mining process. A substantial number of enterprises utilize wheeled machines to perform ore haulage, especially haul trucks and loaders. These machines’ work consists of repeating cycles, and each cycle can be divided into 4 operations: loading, driving with full box/bucket, unloading and driving with empty box/bucket. Monitoring this process is essential to create analytical tools that support foremen and other management crew in achieving effective and optimal production and planning activities. Unfortunately, information gathered regarding the process is frequently based on operators’ oral testimony. This process not only allows for abuse but is also a repetitive and tedious task that must be performed by foremen. The time and attention of foremen is valuable as they are responsible for managing practically everything in their current mine section (machines, operators, works, repairs, emergencies, safety, etc.). Therefore, the automatization of the described process of information gathering should be performed. In this article, we present two neural network models (one for haul trucks and one for loaders) build for detecting work cycles of the ore haulage process. Both models were built utilizing a 2-stage approach. In the first stage, the models’ structures were optimized, while the second was focused on optimizing hyperparameters for the structure with best performance. Both of the proposed models were trained using data collected from on-board monitoring systems over hundreds of the machines’ work hours and utilized the same input features: vehicle speed, fuel consumption, selected gear and engine rotational speed. Models have been subjected to comprehensive testing during which the efficiency and stability of the model responsible for haul trucks was proven. Results for loaders were not as high quality for haul trucks; however, some interesting facts were discovered that indicate possible directions for future development.

## 1. Introduction

Nowadays, new technologies are being introduced to various environments as Industry 4.0 progresses, and the mining enterprises are no exception [1]. A large part of this revolution consists of the introduction of automated industrial process monitoring techniques [2]. Mining enterprises have numerous large-scale, complex processes that can or are already benefitting from the introduction of automated monitoring. This trend has increased to the point where fully autonomous drones are being tested for possible implementation in inspection procedures [3]. This research is focused on one such automation ready process, i.e., monitoring ore haulage. This task mainly consists of transporting excavated material from site A (usually the mining face) to site B (commonly a grid/crusher). The means of transportation used to fulfill this duty largely depends on the infrastructure that is implemented in selected mine; however, this research was conducted for wheeled machines, specifically the LHD machines (Load Haul Dump machines, known also as loaders) and haul trucks. Each machine’s haulage work is divided into a set of repeating operational cycles, and all of the information about this process generally comes from statements by the operators or foreman, meaning that new technology offers opportunities to improve the process.

The work cycles for each machine type can be divided into 4 components: driving with empty cargo box/bucket, loading, driving with full cargo box/bucket and unloading. The problem of automated monitoring of ore haulage work cycles (along with cycle components) for wheeled machines is not new, and some approaches can be found in the literature. These methods can be divided based on two criteria: by monitoring the vehicle and by the time series used for the algorithms. The easiest way to detect the haulage cycles is to monitor the hydraulic pressure measured at the hydraulic cylinder (bucket/cargo box), where the cycles components are clearly visible. Such an approach was presented by the authors of [4], where repeated convolution with reversed unit step were used to detect work cycles along with cycles components for loaders. The algorithm showed an accuracy of 96.3% for two cycle components (driving empty/full) and 67.4% for the third (unloading). The results were calculated based on 300 cycles sample. Another approach using the loader’s hydraulic signal was presented in [5], where authors used Kalman filtering to smooth the signal and a simple thresholding procedure along with validating the work cycle time duration. A continuation for this work was presented later in [6], where statistic methods based on the characteristics of cycle components were introduced. Authors declared the accuracy of their newer model to be 74.4% for unloading, 75.9% for driving with full bucket and 80.19% for driving with empty bucket, all of which were calculated based on a 2-day sample of the loader’s work. They also developed various methods of signal smoothing to test and improve the results [7,8]. Unfortunately, monitoring the pressure signal of the working system’s hydraulics is problematic for both haul trucks and loaders. The sensor itself is known to frequently malfunction and it is hard to implement for machines already in use; therefore, it is being implemented less frequently each year [9,10].

Some methods can also be found to estimate work cycles without the use of hydraulic pressure signals; those are mostly (if not fully) dedicated to haul trucks. Authors of [11] presented an algorithm based on thresholding along with statistical criteria that used information about the selected gear, machine speed, braking system pressure and engine rotational speed. This algorithm scored 90% accuracy on a sample of 93 cases. A similar approach was presented in [12], but it only used the braking system’s pressure signal and the engine’s rotational speed. Unfortunately, the authors did not provide efficiency metrics. A different approach was presented in [13], where authors used machine speed (smoothed with moving average), engine rotational speed and an artificial logic signal created from merging the two. Those three signals were then used to feed the Support Vector Machine (SVM) algorithm along with the DBSCAN algorithm. Such methodology was then validated based on data from 45 days of machine work; however, no efficiency metrics were provided. One of more advanced approaches (from the technical point of view) was described in [14]. The authors used the JADE algorithm on the engine’s rotational speed signal, the signal of instantaneous fuel consumption and machine speed. The components returned from the algorithm were then applied to estimate the kernel density function, which was used for threshold estimation. Unfortunately, the efficiency metrics were not presented.

The literature review uncovers some trends. There are only a few methods of work cycle detection for loaders, and they are all based on the hydraulic pressure signal. One possible reason for this could be that the process itself is especially complicated when considering loaders [15]. On the other hand, there are many more methods regarding haul trucks, and they usually involve using machine speed and engine rotational speed. The common factor between methods is that most of them score accuracy in a way that makes implementation impossible (if it is even described), and/or are validated on rather small samples. In addition, solutions can be found that do not involve using the simple onboard monitoring systems but rather signals such as GPS [16] or vibration measurements [17]. Unfortunately, regardless of their efficiency, they tend to be costly to implement or impossible to incorporate in underground mines.

Although literature for the assumed task is limited (especially when considering loaders), the task of operational regimes detection is not. Expert systems are being constantly created that work based on standard linear programming and logic rules, such as the one presented in [18]. Another trend involving usage of methods from artificial intelligence areas can be observed. Methods utilizing supervised learning, especially neural networks, tend to be more popular [19,20]. However, some unsupervised learning applications can also be found [21]. In this research, the neural network models (deep and convolutional) were utilized both because their usage in the task of haulage cycle identification has not yet been researched and because they tend to perform better than standard approaches [22].

In our research, we wish to present the methodology used to build two neural network models that can be used for work cycles detection. What distinguishes the approach presented in this article and brings some novelty to the already researched topic is as follows:

- both of the models used the hydraulic pressure signal as a reference, but did not use it as input feature (one of the first such approaches for loaders);
- both of the models were fully validated on large scale samples in terms of efficiency and stability;
- results acquired for haul trucks are currently the best available, indicating that the model can be implemented into the information-gathering process;
- results acquired for the loaders, despite being weak for most of the machines, are very good for one (with considerable contribution to sample size). This machine case may pave the way for further research.

The structure of this article is as follows. In Section 2, the ore haulage, machine monitoring system and the methodology of creating the models are described. Section 3 shows the results at each point of creation, validation and testing, along with detailed descriptions of the quantity of data used at each step. Finally, Section 4 states our conclusions.

## 2. Materials and Methods

The ore haulage process can be performed in several ways, and one of them (used for longer haulage routes) is completed using two types of wheeled vehicles: haul trucks and loaders (Section 2.1). For this reason, this research constructed two neural network models for work cycles detection, one for each machine type (Section 3). The data fueling these models were acquired from the machine’s on-board monitoring systems (Section 2.2). Both machine types are equipped with similar measurements systems, so the input variables for the models are identical. The reference signal used for models training also originates in that data, but the specialized algorithms used to obtain the data were different (Section 2.3). Another similarity for both machine types is the model construction process, which can be divided into two general tasks: the optimization of the network structure (Section 2.4) and the optimization of the model hyperparameters (Section 2.5).

### 2.1. Ore Haulage Process

The ore transportation process is the second most important operation in numerous mining enterprises (after ore extraction). This process can be performed using four means of transportation: wheeled vehicles, trains, conveyors (understood as horizontal transport) and shafts (understood as vertical transport). Each mode of transport has different characteristics that correspond to its efficiency, effort of initial implementation, cost of use, etc. [23] In this research, the focus was on wheeled machines used in ore haulage, i.e., haul trucks and loaders. Both machines usually perform spoil transportation between the mining face and the grid. There are two configurations in which this kind of ore transportation can be performed with these types of machinery [24]; by using the loader either alone or accompanied by haul trucks. Work cycles for both machine types are presented in Table 1.

When there is not much material to transport, or when the transportation path is short, the loader can be allocated to complete this task alone. However, most cases require the second configuration, where a single loader is assigned to the mining face along with several haul trucks. In such situations, the loader is responsible for filling up the haul trucks. The overall process is similar to the first configuration, but it is more time efficient. The loader drives to the ore pile, then uses its bucket to raise some of the material. It then drives to the haul truck, where the unloading takes place (bucket is tilted down and then lowered). This whole process represents one work cycle. Three LHD unloadings are usually needed to fill the entire cargo box of the haul truck. Similar operations can be determined for haul trucks. Their work cycle begins with driving (cargo box empty) to the loading site, where the cargo box will be filled by the designated loader. Once full, the haul truck will drive to the site where it will perform unloading (either by tilting the cargo box or by pushing the spoil out of it).

Because the number of performed work cycles and other important KPIs are being calculated based on oral testimony, risk of abuse is introduced to the system that can potentially disturb the reports. Furthermore, requesting the information manually means that companies are unable to adequately estimate some KPIs that are important to the process, such as cycle time [25] or energy efficiency [26]. One possible solutions is the use of machine monitoring systems to estimate work cycles automatically.

### 2.2. Input Data

Nowadays, it is common for the machines that are used in the mining industry to have some form of monitoring system. Depending on the machine and the company, such systems can measure a variety of signals, which provide more or less information. Data in this research comes from an on-board monitoring system which measures over a dozen variables, where most of them are consistent between loaders and trucks. Each of the signals is first measured with frequency of 100 Hz; later, the archiving system automatically calculating the mean value aggregated to 1 Hz. Access to larger sampling in this case is possible but very limited, as it requires a worker to manually download the data. Therefore, the 1 Hz data were used as they allow for complete automation.

As mentioned earlier, the indirect variable used as a reference for training the models is the hydraulic oil pressure signal from the working system (cargo box for trucks, bucket for loaders). Authors of [27] analyzed which other variables should be used for the task of detecting work cycles. The result of their research was the selection of three most important variables: SPEED, SELGEAR and FUELUS. Based on our initial results and other approaches, we added the engine rotation signal to the list. This resulted in the following signals being utilized:

- SPEED—Instantaneous machine speed [km/h];
- SELGEAR—Selected gear of 9 possible [–4, –3, –2, –1, 0, 1, 2, 3, 4], where positive value means driving forward and negative means driving backwards;
- FUELUS—Instantaneous fuel consumption [l/h];
- ENGRPM—Engine rotational speed [rpm];
- HYDOILP—Hydraulic oil pressure of working system [kPa] (reference);

The above-mentioned list of variables was chosen not only because their impact and correlation with HYDOILP has been proven. They are also all of very good quality, and they are the highest of all signals measured in terms of missing values, sensors MTBF (Mean Time Between Failures) and availability. In addition, almost all mining machines utilize some form of sensory network which usually covers the presented list.

**Figure 1:**
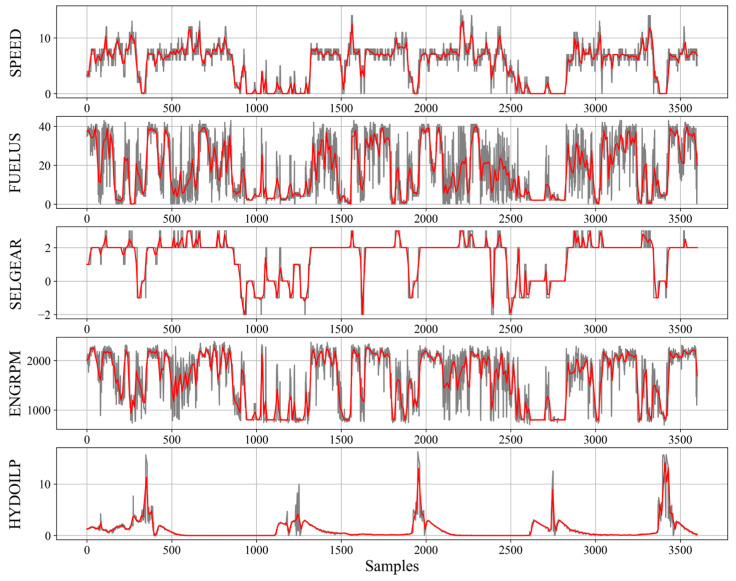
Raw and averaged waveforms of variables used in detection of work cycles. Example generated on data from haul truck.

The method of input data processing, their normalization and structure are crucial for the neural network model effectiveness and stability [28]. Each input data chunk consisted of 4 signals describing one hour of machine work. Their preprocessing was focused on filling missing values with forward fill technique and scaling all variables with the use of their maximum and minimum values (min-max scaler). In addition, the moving average (with 10 s wide window) was applied to each signal, which reduced overall input sample shape from 4x3600 to 4x360 without making a significant impact on effectiveness. The averaging operation was completed only for haul trucks because their cycles duration is much longer than the loaders’ cycles. Raw signals along with their averaged equivalents are shown in Figure 1.

The described preprocessing method that was used to create the input samples was the same for both haul trucks and bucket loaders (with the exception of the moving average). The differences appeared later (Section 2.3) in form of the method used to estimate the reference variable during models training (haulage cycle detection).

### 2.3. Work Cycles Reference Signal

**Figure 2:**
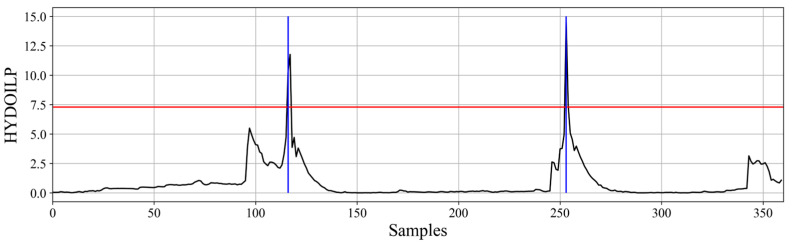
Signal of hydraulic oil pressure from cargo box unloading system for haul truck along with detection threshold (red) and detected unloading moments (blue).

The hydraulic oil pressure signal allows for estimation of work cycles in case of both haul trucks and bucket loaders. However, the variable itself along with the estimation process is drastically different depending on the machine type. In case of haul trucks, the only information that can be extracted is the moment of unloading that appears as a large peak in the pressure signal (matching the cargo box operation). Therefore, the algorithm for their detection is quite simple and involves using a threshold to divide the signal into two values: unloading and other. This is shown in Figure 2.

**Figure 3:**
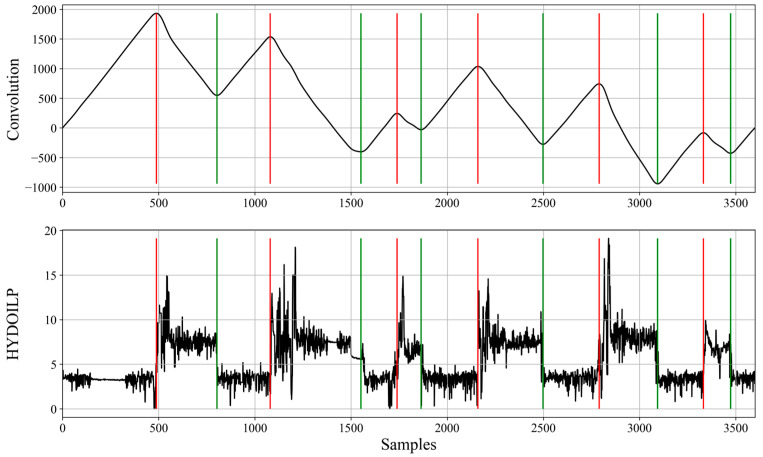
The convolution signal of the HYDOILP variable with unit jump that was used for cycles detection along with the raw HYDOILP variable and critical moments marked (unloading—green, loading—red).

For bucket loaders, the HYDOILP variable is more informative and allows for detection of each of the work cycle components. This estimation can be performed using the algorithm presented in [4], but we decided to use a modified (shortened) version of it. The cycle detection is performed by finding the local extrema moments on the convolution signal between the HYDOILP variable and the unit jump function. Such extrema points corresponded to loading and unloading moments; the introduced modification stopped the algorithm after their detection. This action resulted in detection of three values: loading, unloading and other. This procedure is shown in Figure 3.

Initial trials showed that the categorization approach is much more effective than the regression. As each of the input samples consists of several work cycles, it was decided to split it further using a windowing operation. Window length differed depending on the machine type (mainly because of the operation schema): 30 s for loaders and 120 s for haul trucks. Therefore, for each input sample of shape 30x4 for loaders and 12 × 4 for trucks, the model returned the category vector of length 2 for trucks and 3 for loaders (resulting values were encoded with one-hot method).

### 2.4. Selection of Model Structure

Neural network structure is determined by the order and appearance of layers inside the model. Usually, frameworks that are used for the construction of neural networks have some pre-defined layers that are used as building blocks. This is essentially the most important factor that needs to be faced when constructing NN models [29,30]. Although the usage of layers leaves significant freedom in designing the structure, some predefined standards have been established, such as deep neural networks, LSTM (Long Short-Term Memory) networks, recursive neural networks and others [31]. In this research, two general architectures have been considered: deep and convolutional neural networks. Deep NNs are built mainly using dense layers, while the convolutional NNs have convolutional layers along with at least one dense layer. A possible dropout layer was added to this two-element set of possible layers to oppose possible overfitting and increase model generalization ability.

Choosing the right network structure for the model significantly increases the results achieved with it. From all the possible layer combinations, manually constructing the network structure is a tremendous task that will most likely fail to achieve the most optimal arrangement. Therefore, the following algorithm, which is rather simple but effective, was implemented to automate this process:

1. Create a space of all possible structures, within the assumed parameters;
2. Remove from that space all of the structures that are incorrect or incompatible with two general architectures;
3. Pre-train all structures with default hyperparameters and evaluate their performance;
4. Return one best structure.

The structures space was initially created with two main boundaries: 1—all structures need to be built from the combination of 3 layers: convolutional, dense and dropout, and 2—there was a maximum number of layers set that the structures can achieve. The space then was crafted by iteratively taking Cartesian product of two sets of layers combinations as it is showed in below formulas (1) and (2).

(1) $$C=\sum_{i=0}^{n}X_{i}$$

(2) $$X_{i}=\left\{\begin{array}{cc} X_{i-1}\times A, & i>1\\ A\times A, & i=1\\ A, & i=0 \end{array}\right.$$

where C is a set containing all possible combinations of neural network structures up to maximum number of layers $\left(n\right)$, $X_{i}$ denotes all possible structures for specified number of layers $\left(i\right)$, and A is a starting build block, $A=\left\{dense,\;convolutional,\;dropout\right\}$.

When the structures space was created, some of the combinations had to be removed, mainly because of their incorrectness or incompatibility. Structures that did not meet the following criteria were removed:

- All of the convolutional layers in the network must form a consistent block in the beginning of the structure, and can be only intertwined by dropout layers;
- The Structure has to possess at least one dense layer;
- There cannot be two sequential dropout layers in a structure.

After cleaning of the space, the structures were ready to be trained. The default hyperparameters used in this process were: 360 neurons and ‘relu’ activation function for dense layers, 16 filters and (3, 3) filter size for convolutional layers, 30% probability for dropout layers, categorical cross-entropy loss function and Adam optimizer with default settings. The training process of the networks was handled by using the EarlyStopping callback, set on parameters that ensured faster but less accurate training (pretraining). Finally, all the trained networks were evaluated, and the best network was chosen.

### 2.5. Hyperparamters Optimization

Hyperparameters of neural network model layers are the second most important factor, after the model’s structure. The task of their optimization is usually performed in a similar way to the process presented for structure optimization. The key difference is that the optimization algorithm operates in param space instead of a structure space. The Talos library [32] was used to optimize models’ hyperparameters in this research. This library supports all of the popular neural network frameworks (tensorflow, pytorch, keras).

The optimization of hyperparameters with the Talos library began with declaration in the form of a dictionary. The keys of that dictionary corresponded to hyperparameters, and the possible parameter values were stated for each key. Using this dictionary, the param space was created (again with by using the Cartesian product). For this optimization task, the hyperparameter keys used along with their description are presented in Table 2.

After creation of param space, the Talos library performed the optimization by iteration through all param space elements. Each of the possible combinations were created using the dictionary keys, then trained and finally evaluated.

## 3. Results

The main result of the research presented in this article was the development of two neural network models with proven efficiency and stability. To achieve this task, methods presented in Section 2 were used on data from different machines and time periods. The amount of data used in this process covered 17,341 h of work from 8 haul trucks and 16,812 h of work from 8 loaders. However, due to large class imbalance, the data used were converted to samples and then downsampled to match the length of least occurring class. This procedure largely reduced the number of analyzed work hours, but the change was necessary as the class imbalance was as high as 20 to 1 in some cases. Therefore, the final numbers of samples used to create models were:

- 64,288 2-min samples for haul trucks, 31,113 per class (2 classes), resulting in a total of 2143 h of work;
- 102,042 30-s samples for loaders, 34,014 per class (3 classes), resulting in a total of 850 h of work.

This data was then used in the optimization process for both structure and hyperparameters (Section 3.1 and Section 3.2 respectively), during which two optimal models were found and created. Next, the stability of the models was tested (Section 3.3), and finally the results for overall sample were acquired (Section 3.4).

### 3.1. Results of Structures Optimization

Because the task of cycles detection is more difficult for loaders (3 classes and different work schema), it was assumed that the model should be larger for them. Therefore, in optimization for structure, the only optimization criterium (maximum number of layers) was set to 10 for loaders and 8 for haul trucks. Both parameters are assumed to be without input and output layer.

**Figure 4:**
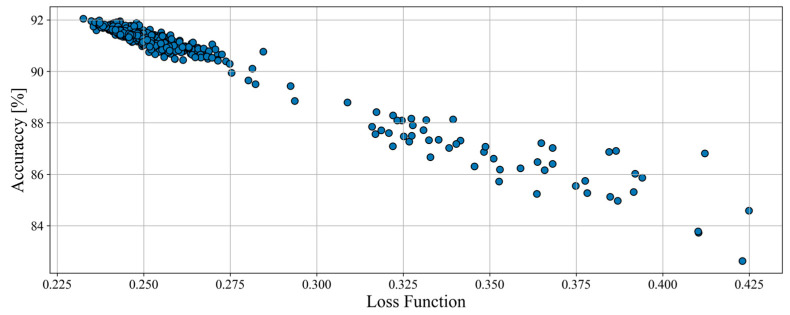
Accuracy and value of loss function of structures examined during the structure optimization process for haul trucks.

The total amount of tested structures for haul trucks was 477, and their results (on the plane of accuracy and loss function) are showed in Figure 4. None of the models scored an accuracy of less than 80% which proves that the data processing and overall assumptions are good.

**Figure 5:**
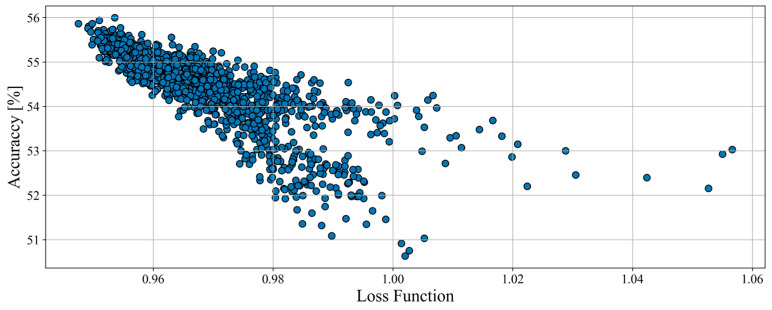
Accuracy and value of loss function of structures examined during the structure optimization process for loaders.

The total number of tested structures for loader was 1585, almost 3 times higher than for haul trucks. However, the results obtained (Figure 5) were significantly worse. The vast majority of networks were only between 51% and 57% accurate. The results, although worse, are still better than random hit probability (33% for 3 classes).

The following two structures were obtained in the process of structure optimization:

- For haul trucks: Input Layer, Convolutional, Dropout, Flatten, Dense, Dense, Dropout, Dense, Output Layer;
- For loaders: Input Layer, Convolutional, Convolutional, Convolutional, Convolutional, Dropout, Flatten, Dense, Dropout, Dense, Dense, Output Layer.

Structure for haul trucks scored the accuracy of 92.02448% and loss value of 0.232505, while the structure for loaders scored respectively 56.9949% and 0.953545.

### 3.2. Results of Hyperparameters Optimization

Similar to the task of structure optimization and based on its results, the possible parameter space for loaders was extended. Parameters used in this optimization for each machines type are presented in Table 3.

**Figure 6:**
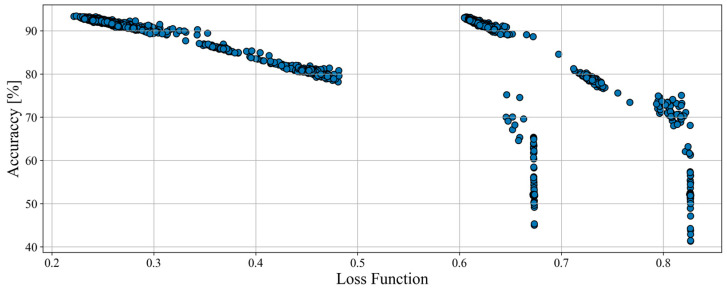
Accuracy and value of loss function of params combinations examined during the hyperparameters optimization process for haul trucks.

The total number of tested combinations for models hyperparameters was 2592 for haul trucks, and its results are shown in Figure 6. During this optimization, only a small number of networks showed improvement of results, which suggests that the initial hyperparameters chosen for structure optimization were optimal. This can also indicate that better results may not be achievable for haul trucks (for whatever reasons). Another item to be observed is that the results clearly form clusters and shapes. Results appear to be merging and forming line patterns.

**Figure 7:**
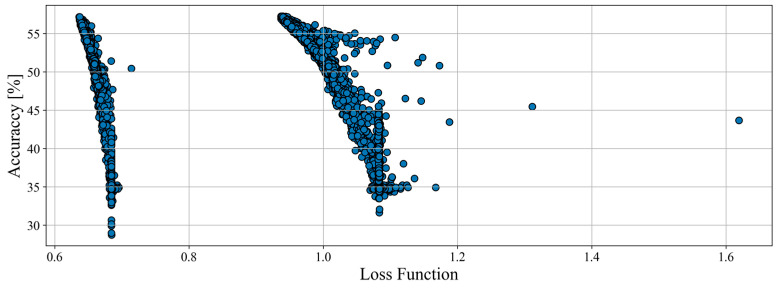
Accuracy and value of loss function of params combinations examined during the hyperparameters optimization process for loaders.

The total number of tested combinations for model hyperparameters was 10,368 for loaders, and its results are shown in Figure 7. Even though the param space being 4 times larger, a situation similar to haul trucks is visible for the loaders. Only a small number of the sample showed some improvement, and none of the networks scored more than 57% accuracy. The clusters are visible again; however, their behavior is different. Instead of multiple dense clusters, the results formed 2 large and more disperse ones.

**Figure 8:**
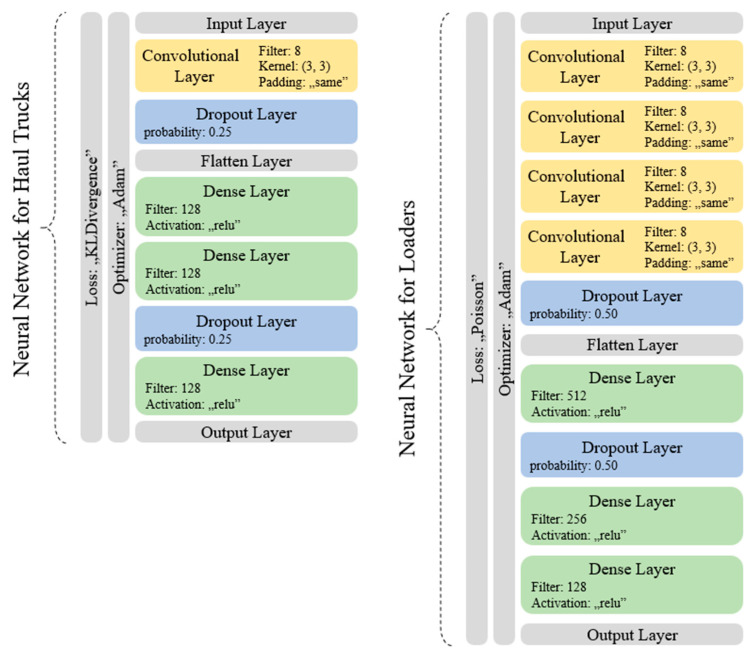
Final structures and hyperparameters of both neural networks acquired during the optimizations described in construction process.

Optimization of hyperparameters was the second and last step of model’s creation. During this process, the final structures presented in Figure 8 were acquired. The model for haul trucks scored an accuracy of 93.4107% and a loss value of 0.223873, while the model for loaders scored 57.1791% and 0.636359, respectively.

**Figure 9:**
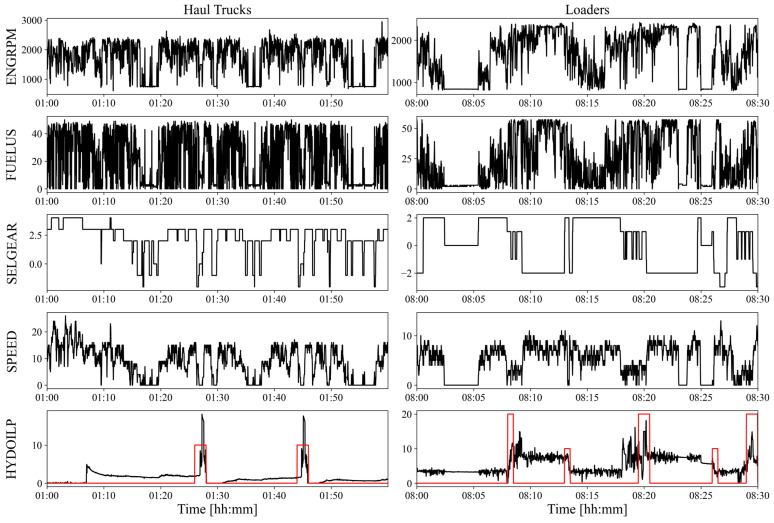
Examplary detection performed by model for haul trucks (**left**) and by model for loaders (**right**). Each detection is presented as a red line (scaled by 10 to be visible) presented at HYDOILP variable. All variables presented are raw.

Exemplary detection performed by final models can be seen on Figure 9. The figure shows a set of raw input variables for both models (ENGRPM, FUELUS, SELGEAR and SPEED) along with the reference variable (HYDOILP), which was also used to present the detected cycles (red line). For better presentation, a different chart length is used for both machines, with 1 h for haul trucks and 30 min for loaders.

To summarize, the process of hyperparameters optimization showed that the selection of initial networks parameter was optimal, and that the emphasis should be put on the network structure (in this particular task). Regardless of the amount, some improvements were achieved in this process. The network for haul trucks scored about 1.3% higher accuracy while the network for loaders improved by about 0.3%.

### 3.3. Testing the Stability of Models

**Figure 10:**
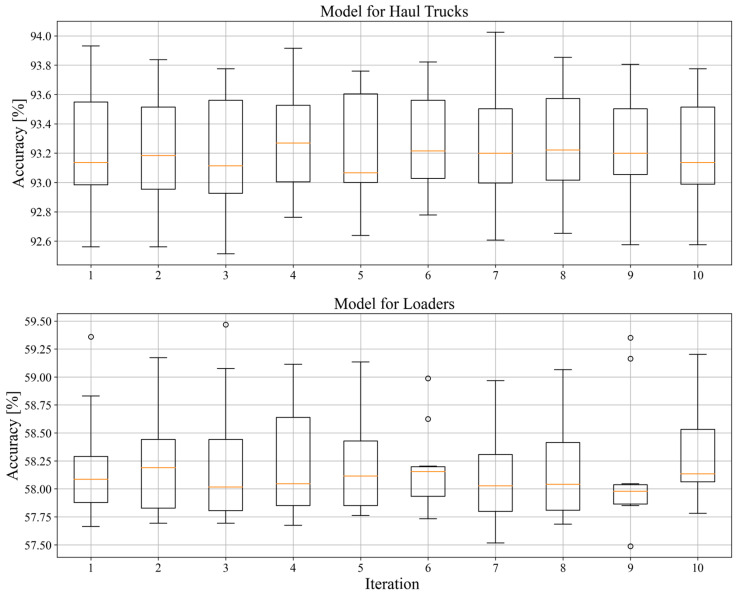
Results obtained from testing the stability of the models using the modified k-fold cross validation algorithm (Upper—network model for haul trucks, Lower—network model for loaders).

To establish the stability of models, the modified k-fold cross-validation algorithm was used. In its original form, this algorithm divides all data into *k* parts and performs *k* learning processes, during which each of the *k*-1 parts are used for training and the remaining part is used for validation. The one validation part is changed during each training process until none of the *k* parts are used for that purpose. In the modified version used for this research, each of the *k* learnings is repeated *n* times (with the same data division), to focus on the randomly initiated networks parameters such as weights and kernel filters. For both models, values of *k* = 10 and *n* = 10 were used, and the results from that procedure are shown in Figure 10.

The model for haul trucks showed high stability, efficiency and accuracy with mean values in the range of 93.0%–93.4%, with the highest score being 94.0239% and 92.5135% as the lowest score. Unfortunately, the model for loaders showed none of the predecessor’s quality, with mean values in the range of 57.75%–58.25%, with the highest accuracy being 59.4672% and lowest one being 57.4876%.

### 3.4. Results Achieved by Models

To assess the overall results of the models, two methods of analysis were selected: confusion matrix and accuracy analysis relative to the machines measurements proportion in general sample size. The model for the haul truck was built to detect two states, and its confusion matrix is showed in Table 4. The results are good and show that the network model is slightly better at detecting the unloading operation. It can also be seen that the network in general tends to declare an unloading state rather than the other state, although this difference is minimal (up to 3%).

The confusion matrix for loaders is presented in Table 5, and is respectively larger as the model was built to detect three different states instead of two. Matrix show that the networks perform the worst when trying to detect unloading operation, which is most frequently confused with loading operation. It was partially to be expected as these two operations are similar to each other in terms of signals other than hydraulic signal from working system. Both operations possess similar movement pattern during which higher engine rotational speed occur that causes higher fuel consumption. Unfortunately, the confusion matrix continues to confirm that the model for loaders has room for improvement.

**Figure 11:**
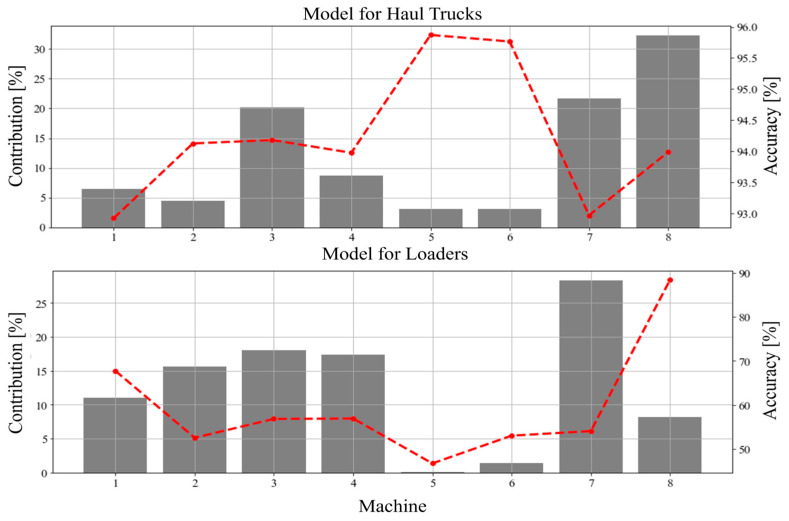
Accuracy (red line) for data from machines in relation to their contribution (gray bars) to the overall sample size (Upper—Haul Trucks, Lower—Loaders).

For the second analysis, all data were grouped in datasets based on unique machines (1 dataset—1 machine). Accuracy achieved for these datasets, along with their size in relation to all possessed data, is showed in Figure 11. For haul trucks the analysis further confirms the overall good results of the model. The instability in accuracy depending on the machine used in this case is low (below 3%). For the loaders, however, the analysis shows some interesting facts. The primary item of note is that the accuracy as scored by the model was close to 90% for one machine. Considering the appropriate size of data from this machine in the sample, the suspicion that it is an accident or an outlier can be dismissed. As for other machines, the network shows a large instability in accuracy, around 30% when the machine no. 8 is not considered.

Finally, to summarize the achieved results, we decided to perform a comparison between effectiveness of models created in this research and results declared by other authors (presented in Table 6). Unfortunately, many approaches do not clearly state their achieved performance, thus, only 3 other models were included in this comparison. Accuracy for the presented approach was established based on the confusion matrices as mean accuracy from sets. All metrics were normalized to a range from 0–100% in order to be comparable. As can be seen, the presented models are innovative in terms of sample size, utilized variables and detected regimes. Regarding the haul trucks, there is also an improvement in accuracy.

The overall results are different for both machine types. The neural network model created for haul trucks shows undeniable signs of stability along with performance. Accuracy achieved by this model is independent from data collection, random initial state and machine. These factors indicate that the model can be implemented and actively used in mining enterprises. The opposite is represented by the model constructed for loaders. Results obtained from this model are mediocre show signs of instability. Therefore, it cannot be recommended for implementation.

## 4. Conclusions

Automated process monitoring is an important factor for predictive maintenance, operational assessment and others. This article presented the construction method of a neural network for detection of work cycles in haul trucks and loaders that carry out ore haulage. The main goal of this article was to present models that are independent from the hydraulic oil pressure signal because the signal fails frequently and is not always available. As a result, two neural network models were created, one for each machine type. The process of each model creation was divided into two steps: optimization of model structure (order of the network layers) and optimization of structure hyperparameters.

Constructed models were fueled using data from on-board monitoring system of vehicles (which is consistent between machine types). Both models used the same set of input features: instantaneous vehicles speed, fuel consumption, engine rotational speed and selected gear. A hydraulic pressure signal was used as a reference in training and validation, processed by different algorithms depending on machines type. The models were trained to perform a task of classification, and therefore the model for haul trucks is able to distinguish between two operations: unloading and other. The model for loaders supports one more cycle stage: unloading, loading, other.

The data initially provided for this research consisted of 17,341 h of work for 8 haul trucks along with 16,812 h of work for 8 loaders. Since most of the hours did not contain a proper reference variable, and later because of large class imbalance, we decided to downsize the samples created to match the least occurring class. Therefore, the final data consisted of 2143 workhours for haul trucks and 850 workhours for loaders. These data were used for training, validation and testing.

The final results were assessed by the k by n fold cross validation (modified k-fold cross validation) algorithm. The model for haul tracks scored a mean accuracy of 93% and was proven stable in multiple assessment tasks. The score for this model along with the size of its sample is currently among the best described in related literature. Unfortunately, the model for loaders did not share the same results; it scored only 58% of accuracy. Despite poor global results for loaders, one machine scored 90% accuracy, which is promising. However, it should be stated that the task of work cycle estimation is much more difficult when it comes to loaders, and the attempt presented in the article was one of the first.

By comparing the results acquired by presented models with those found in the literature, some fundamental facts were established:

- The model for haul trucks acquired the highest accuracy and was trained and validated based on the largest (and possibly the most various) data sample.
- The model for loaders is the first described approach of work cycle estimation based on signals other than hydraulic oil pressure. It was trained and validated based on largest sample. Its accuracy is generally lower in comparison with the rest of approaches, except for the machine 8, for which it is higher than mean general accuracies.

Implementation of the haul trucks model will result in noticeable reduction of foremen workload. Assuming that the enterprise is large, each foreman needs to manually request information about machines’ work cycles from up to 20 operators, and then use it to complete the corresponding forms. Automation of this process can save as much as 30 min of work time. Therefore, assuming that large mines employ over a dozen foremen on each work shift, the savings can be measured as several hours per shift. In addition, cycle detection performed using the presented model can be used as data preprocessing stage for predictive maintenance algorithms. One of such approach is presented in [33], where cycle detection was performed by hand.

It is also worth mentioning that, apart from the overall bad results, the model for loaders scored accuracy close to 90% for one machine while having a not small share of the sample. Investigating this outlier along with an attempt of transfer its success to other loaders is a future direction of research.

## Figures and Tables

**Table 1:** Components of a single haulage cycle for loaders and haul trucks.

No.	Loader	Haul Truck
1.	Driving with empty bucket to the mining face	Driving with empty cargo box to mining face
2.	Loading and raising the bucket	Cargo box expanding (if necessary) and loading
3.	Driving with full bucket to the haul truck or grid	Driving with full cargo box to the grid
4.	Unloading and lowering the bucket	Cargo box unloading (by tilting or sliding)

**Table 2:** Hyperparameters of the optimized networks.

Hyperparameter	Description
conv2d_number	Number of convolutional filters in each of the convolution layers (same for each layer)
conv2d_size	Shape of the convolutional filters used in convolution layers (same for each layer)
dens_neurons	Number of neurons used in the first dense layer
dens_divider	Factor specifying how much smaller will be the successive dense layers(applies to all dense layers except the first one)
dens_activation	Activation function in all the dense layers (same for each layer)
dropout	Fraction of input nodes to drop in dropout layers (same for each layer)
loss	Loss function used in the training of the model
optimizer	Optimizer used for the model training

**Table 3:** Hyperparameter values tested during optimization process for haul trucks and loaders.

Parameter	Possible Values for Haul Trucks	Possible Values for Loaders
conv2d_number	32, 16, 8	64, 32, 16, 8
conv2d_size	(2, 2), (3, 3)	(2, 2), (3, 3)
dens_neurons	128, 64, 32, 16	512, 256, 128, 64, 32, 16
dens_divider	1, 2	1, 2
dens_activation	relu, tanh, sigmoid	relu, tanh, sigmoid
dropout	0.25, 0.50	0.10, 0.30, 0.50
loss	CategoricalCrossEntropy, Poisson, KLDivergence	CategoricalCrossEntropy, Poisson, KLDivergence
optimizer	Adam, SGD, RMSprop	Adam, SGD, RMSprop, Ftrl

**Table 4:** Confusion matrix created for haul trucks network model.

$y_{true}$	$y_{pred}\;\left(Training\right)$		$y_{pred}\;\left(Validation\right)$		$y_{pred}\;\left(Testing\right)$	
	Unloading	Other	Unloading	Other	Unloading	Other
Unloading	48.95	0.92	47.54	2.64	47.65	2.49
Other	3.83	46.26	5.55	44.25	5.58	44.26

**Table 5:** Confusion matrix created for loaders network model.

$y_{true}$	$y_{pred}\;\left(Training\right)$			$y_{pred}\;\left(Validation\right)$			$y_{pred}\;\left(Testing\right)$		
	Other	Loading	Unloading	Other	Loading	Unloading	Other	Loading	Unloading
Other	20.99	6.91	5.33	20.01	7.51	5.75	19.77	8.00	5.86
Loading	5.15	22.51	5.48	5.66	20.81	7.30	5.46	20.78	7.13
Unloading	7.68	7.55	18.34	9.32	9.32	15.30	8.09	9.15	15.71

**Table 6:** Comparative analysis of results between models presented in this article and other models in literature which accuracy is known.

	Haul Trucks	Haul Trucks	Loaders	Loaders	Loaders
Parameter	Presented Model	Krot, et al. [11]	Presented Model	Koperska, et al. [4]	Polak, et al. [6]
Accuracy—total [%]	92.9	90.0	58.2 (90% for machine 8)	86.6	76.8
-Unloading	96.0	90.0	49.5	67.4	74.4
-Loading	89.8 (for loading, driving full and empty)	-	64.2	-	-
-Driving Empty		-	60.9 (for driving full and empty)	96.3	80.2
-Driving Full		-		96.3	75.9
Sample Size	2143 h	93 work cycles (~20 h)	850 h	300 work cycles (~60 h)	2 days (~12 h)
Variable Utilizing	speed, engine rotation, fuel consumption, selected gear	speed, selected gear, engine rotation, brake pressure	speed, engine rotation, fuel consumption, selected gear	hydraulic oil pressure	hydraulic oil pressure

## Data Availability

Not applicable.
